# The Mental Health and Well‐Being of Adults With Intellectual Disabilities During the COVID‐19 Pandemic Across the UK: A Four‐Wave Longitudinal Analysis

**DOI:** 10.1111/jir.70006

**Published:** 2025-07-15

**Authors:** Amanda Gillooly, Paul Thompson, Jill Bradshaw, Sue Caton, Chris Hatton, Andrew Jahoda, Rosemary Kelly, Roseann Maguire, Edward Oloidi, Laurence Taggart, Stuart Todd, the Coronavirus and People With Learning Disabilities Study Team, Richard P. Hastings

**Affiliations:** ^1^ School of Mental Health and Wellbeing University of Glasgow Glasgow UK; ^2^ Intellectual Disabilities Research Institute University of Birmingham Birmingham UK; ^3^ Department of Social Care and Social Work Manchester Metropolitan University Manchester UK; ^4^ School of Nursing and Midwifery Queen's University Belfast Belfast UK; ^5^ Faculty of Life Sciences and Education University of South Wales Pontypridd UK

**Keywords:** COVID‐19, growth models, intellectual disability, mental health, well‐being

## Abstract

**Background:**

Research concerning the impact of the COVID‐19 pandemic on the mental health and well‐being of adults with intellectual disabilities has been cross‐sectional and small scale. We examined the trajectory of mental health and well‐being across the pandemic period across the UK and the factors which predicted different mental health trajectories.

**Method:**

Adults with intellectual disabilities participated in co‐designed structured interviews. Four waves of data were collected between December 2020 and late 2022. At Wave 1, 621 adults with intellectual disabilities participated, with 355 at Wave 4. Well‐being, pandemic anxiety, depression, anxiety, anger and loneliness outcomes were measured. Latent class mixed modelling was used to identify subgroups and within‐group trajectories.

**Results:**

Well‐being and pandemic anxiety remained relatively stable across time, but levels of anger, depression, anxiety and loneliness reduced gradually over time. Overall patterns masked trajectory subgroups, with differences in intercept and steepness of decline or increase in mental health problems. Different factors were generally influential for trajectory class membership and overall change across time for outcomes. Leaving the house for exercise or green spaces reported increasing well‐being and reduced loneliness. Similarly, those working, volunteering or in education at Wave 1 were found to have increasing well‐being and reduced loneliness, sadness and worry, and increasing wellbeing and reducing anger if they were working pre‐pandemic.

**Conclusions:**

Social connection and engagement in purposeful activity were vital to maintaining the mental health and well‐being of people with intellectual disabilities. Factors that were found to reduce mental well‐being during the pandemic should be considered in planning for future major public health challenges and in promoting better mental well‐being for people with intellectual disabilities in everyday life.

Although people with intellectual disabilities experience significant mental health inequalities (Cooper et al. 2015), they have inequitable access to appropriate intervention and support, such as psychological therapies (Whittle et al. 2018). Systemic threats to the mental health and well‐being of people with intellectual disabilities increased with the onset of the COVID‐19 pandemic and associated public health restrictions. Negative impacts included increased social isolation, reduced access to health and social care services and the reduction or withdrawal of social and community supports such as day services (Flynn et al. 2022; Hatton et al. 2024; Rosencrans et al. 2021).

A thematic synthesis of 20 qualitative and mixed method studies exploring the experiences of people with intellectual disabilities during the COVID‐19 pandemic (Parchomiuk 2024) showed that researchers largely used proxy reports by family carers or professionals and evidenced a multitude of reported negative impacts on the mental health of people with intellectual disabilities. Deteriorating mental health was often linked to a loss of routine, less social contact with family and friends and reduced engagement in meaningful activities. We found only one quantitative study including longitudinal data on the mental health and well‐being of adults with intellectual disabilities. Totsika et al. (2021) analysed data from two UK cohort studies of 20‐ and 50‐year‐olds, including pre‐pandemic data and data gathered in May 2020 using a special COVID‐19 survey supplement. The analysis focused on May 2020 mental health differences between adults with intellectual impairment (IQ > 1 SD below the mean for the sample) and comparison adults. After controlling for pre‐pandemic levels of mental health, gender and ethnicity, there were no group differences in reported poor mental health for the 20‐ or 50‐year‐olds. Although pre‐pandemic data were available, the reported analysis did not consider pre‐pandemic versus May 2020 levels of poor mental health in the intellectual impairment sample alone.

While cross‐sectional and qualitative evidence supports a hypothesis that there were negative effects of the pandemic on the mental health of people with intellectual disabilities, there are significant methodological limitations to the current evidence and a lack of longitudinal research. Most of the quantitative research has been small scale and cross‐sectional in design, with very few studies reporting data directly from people with intellectual disabilities. The present study addresses these limitations and extends research over multiple time points throughout the pandemic as threats and public health restrictions changed. A longitudinal design provides a unique opportunity to obtain a more nuanced understanding of the factors that increased individuals' vulnerability to mental health problems across time or factors that were protective for individuals.

Several key protective factors have been shown to maintain mental well‐being in people with intellectual disabilities, including community and social supports, physical exercise and an engagement in meaningful activities (Diz et al. 2024; Parchomiuk 2024; Stancliffe et al. 2007). These were the focus of this study. We investigated how the mental health of adults with intellectual disabilities changed across the different phases of the COVID‐19 pandemic across the UK; how the variables age, gender, living circumstances, shielding, going out for exercise, support, community activities and involvement in work or further education predicted different mental health trajectories for adults with intellectual disabilities across the COVID‐19 pandemic; and which of these variables predicted changes in trajectories.

## 1 Method

### 1.1 Design

Data were drawn from the longitudinal Coronavirus and people with learning disabilities study (Flynn et al. 2021), which recruited individuals with intellectual disabilities across four waves of data collection: during the winter period of 2020/2021 (mainly during COVID‐19–associated lockdowns), across the spring of 2021 (some public health protection measures were being lifted during this period), in late summer 2021 (protective measures had mostly been removed) and summer/early autumn 2022 (all measures had been removed).

### 1.2 Participants

We recruited adults with intellectual disabilities who were able to be interviewed (see Flynn et al. 2021) (see Table 1 for participant descriptives for the 613 individuals at Wave 1).

**TABLE 1:** Participant descriptive statistics at study Wave 1.

	Overall *N*	Mean	SD
Age	607	38.85	13.37

	Overall *N*	*n*	%
Gender	605		
Female		294	49
Male		311	51
Lives with …	612		
Lives alone		189	31
Lives with family		291	48
Lives with others with ID		132	22
Shielding	613		
Yes		117	19
No		496	81
Community activity	611		
Yes		527	86
No		84	14
Leaving home for exercise or visiting green spaces	608		
Yes		436	72
No		172	28
Work/FE/Volunteering	608		
Not in work pre‐pandemic or currently		158	26
In work currently		235	39
In work pre‐pandemic, but not currently		215	35

### 1.3 Measures

#### 1.3.1 Outcomes

Two versions of the Warwick Edinburgh Mental Wellbeing Scale (WEMWBS; Tennant et al. 2007; intellectual disabilities version, Scior et al. 2023) were used (14‐item and seven‐item versions). For Wave 1, the 14‐item version was used, with items rated on a 4‐point Likert scale (‘never’, ‘sometimes’, ‘often’, ‘always). Participants were asked to respond based on the last week. A mean item score was computed where there were three or fewer missing items. One item on this scale had an error in wording and was treated as missing for all participants. For Waves 2, 3 and 4, the seven‐item version of this scale was used, with items rated on a 4‐point Likert scale. These items were a subset of questions from the 14‐item scale. Participants were asked to respond based on the last week. One item was missing for all participants due to the wording error, and a mean item score was calculated only if the other six items were complete. At Wave 4, the wording error was fixed, and so a mean item score was calculated if any six items had been completed. The mean item score was computed for each wave to allow comparison across waves.

The Pandemic Anxiety Scale (McElroy et al. 2020) is a seven‐item scale with items rated on a 4‐point Likert scale (‘This does not apply to me’, ‘Not at all’, ‘A little’, ‘A lot’). A total score was computed for participants with two or fewer missing items. In these cases, missing items were replaced by a mean score for the individual across all other completed items.

For mental health outcomes, we developed four single‐item questions to capture key mental health outcomes (‘how often did you feel lonely with no one to talk to’, ‘how often did you feel worried or anxious’, ‘how often did you feel sad or down’, ‘how often did you feel angry or frustrated’). Participants were asked to respond based on the last 4 weeks. Items were rated on a 3‐point Likert scale (‘never or hardly ever’, ‘some of the time’, ‘often or always’).

#### 1.3.2 Predictors

Seven predictor variables were considered in the analysis, including the age (in years) and gender of the person with intellectual disabilities at Wave 1; whether they were shielding at Wave 1 (the most vulnerable remained in their homes and avoided face‐to‐face contact); whether they reported being involved in community activities before the pandemic; whether they were living in the family home, living alone or living with others with intellectual disabilities; whether they were leaving the house to exercise or visit green spaces at Wave 1; and whether they were attending college or work at Wave 1, had attended college or worked pre‐pandemic but not now, or had never worked or attended college at Wave 1.

### 1.4 Procedure

Researchers interviewed adults with intellectual disabilities by telephone or online. All participants self‐reported their capacity to take part in the interviews and provided informed consent. Adults with intellectual disabilities had the choice to have a supporter present during the interview. During the interviews, trained interviewers provided further clarification on items where required, e.g., repeating questions, while maintaining within standardisation limits. Interviewers emphasised to participants that there were no right or wrong answers and that we were interested in their own experiences of the pandemic. Interviews lasted around 45 min, with short breaks offered as needed.

### 1.5 Statistical Analysis

The analysis aimed to establish if subgroups were present within the cohort, whether they had different trajectories over time for the mental health and well‐being outcomes and whether particular predictors influenced group membership and/or whether they influenced change over time globally across identified subgroups. Sample size precluded exploration of predictors of change in mental health outcomes within each trajectory group and random slopes, hence the focus on predictors of change overall in the sample.

Using data across four time points, a latent class mixed model (LCMM; Proust‐Lima et al. 2009, 2017) was fitted to each outcome to determine whether there was heterogeneity in the sample over time. The model has two components estimated in parallel: a latent class model to establish group membership, and a longitudinal model that establishes predictors of trajectories. Single‐item categorical outcomes use a different link function and hence have additional threshold parameters estimated accordingly. A threshold model (cumulative probit model) describes the correspondence between each level of the categorical outcome and the underlying latent process (Proust‐Lima et al. 2017).

Baseline models for all outcomes contained time as a covariate only and no other predictors of class membership or trajectory. Main analyses contained both predictors of class membership, class‐specific trajectory (time only, but all other covariates are common to all classes) and random intercepts to account for repeated measurement dependency within the models. For each model, we fitted one to five class models and compared the model fit globally using Akaike's information criterion (AIC), Bayes information criterion (BIC), sample‐size‐adjusted BIC (SABIC), entropy and integrated complete‐data likelihood (ICL). The ICL reports the penalised BIC by including an entropy term favouring well‐separated clusters (ICL1 and ICL2 are slightly different versions of the same penalisation; Tomarchio and Punzo 2025). The best fitting models were chosen by looking for the optimum values of fit indices across the nested class models.

The data contained between 7% and 8% missingness in each covariate and up to 22% across each outcome. Therefore, we used multiple imputation using chained equations for all selected main analysis models containing all covariates as a sensitivity analysis.

All analyses were conducted using the R statistical software (Version 4.2.3), with R package lcmm (Proust‐Lima et al. 2017). Multiple imputation was conducted using the R packages ‘mice’, ‘miceadds’ and ‘countimp’ (Van Buuren 2018).

## 2 Results

Means and standard deviations for all time‐varying variables are presented in Table 2. For continuous outcomes (well‐being and pandemic anxiety), scores remained relatively stable across the four waves, whereas all single‐item mental health outcomes showed a general reduction across time.

**TABLE 2:** Descriptive statistics for outcome measures at each wave.

Outcome	Wave							
	1		2		3		4	
	*N*	Mean (SD)	*N*	Mean (SD)	*N*	Mean (SD)	*N*	Mean (SD)
WEMWBS	605	2.95 (0.62)	572	2.99 (0.64)	507	2.97 (0.64)	355	2.99 (0.59)
PAS total score	568	13.95 (3.77)	555	11.46 (3.47)	487	11.47 (3.52)	355	12.65 (3.91)
Lonely	607	1.76 (0.78)	588	1.65 (0.73)	524	1.54 (0.71)	351	1.51 (0.70)
Anxious	609	2.03 (0.77)	589	1.87 (0.75)	525	1.83 (0.75)	354	1.77 (0.72)
Sad	607	1.93 (0.73)	585	1.79 (0.73)	526	1.75 (0.69)	353	1.73 (0.68)
Angry	606	1.91 (0.75)	590	1.71 (0.69)	526	1.70 (0.69)	355	1.63 (0.66)

### 2.1 Well‐Being—LCMM

For the WEMWBS outcome, a two‐class model was selected as the best fitting model in both base and main analysis (including covariates for both class membership and longitudinal elements) models according to the lowest AIC and BIC values (Nylund et al. 2007) (see Table S1 and Figure S1). Class membership was 62.23% of the sample for Class 1 and 37.77% of the sample for Class 2 (Table S2). A baseline model was fitted to model change across time without other predictors included (unconditional model). By examining the models across time only, a change in well‐being was established. Each class had significantly different intercepts (Class 1 had a higher well‐being score starting point), and there was a significant change over time in both classes (Class 1 showed a small reduction, β = −0.05, SE = 0.02, *p* < 0.01; Class 2 showed a small increase, β = 0.11, SE = 0.02, *p* < 0.0001).

When considering predictors of class membership (Table 3), the following were statistically significant: age ( β = 0.03, SE = 0.01, *p* = 0.01), gender ( β = −0.69, SE = 0.23, *p* < 0.01) and who the person with intellectual disabilities lives with (reference category living alone: lived with family, β = 1.07, SE = 0.29, *p* < 0.01; lives with others with intellectual disability, β = 1.16, SE = 0.33, *p* < 0.001). The coefficients from the class membership model describe how a specific predictor increases or decreases the likelihood of being in a specific class compared to a reference class. Males and older people were more likely to be in Class 1 (slightly reducing well‐being over time), as were those living with family or with other people with intellectual disabilities compared to those living alone.

**TABLE 3:** Fixed effects from latent class mixed models (WEMWBS and PAS outcomes).

Parameter	WEMWBS (mean score)				PAS (total score)			
	Coefficient	SE	Wald statistic	*p*	Coefficient	SE	Wald statistic	*p*
**Fixed effects in the class‐membership model**								
Class 1 (intercept)	−0.30	0.69	−0.44	0.66	0.55	1.01	0.54	0.59
Class 1 (shielding)	−0.04	0.35	−0.13	0.90	0.13	0.52	0.24	0.81
Class 1 (community action)	−0.73	0.51	−1.45	0.15	1.12	0.75	1.49	0.14
Class 1 (Age)	0.03	0.01	2.55	**0.01**	−0.03	0.01	−1.87	0.06
Class 1 (Gender)	−0.69	0.23	−3.01	**< 0.01**	0.12	0.33	0.36	0.72
Class 1 (lives with_2)	1.07	0.29	3.65	**< 0.01**	0.57	0.40	1.40	0.16
Class 1 (lives with_3)	1.16	0.33	3.56	**< 0.001**	0.33	0.43	0.76	0.45
**Fixed effects in the longitudinal model**								
Class 1 (intercept)	3.05	0.08	36.02	**< 0.0001**	12.70	0.68	18.77	**< 0.0001**
Class 2 (intercept)	2.12	0.09	22.46	**< 0.0001**	14.09	0.80	17.52	**< 0.0001**
Class 1 (time)	−0.05	0.02	−3.08	**< 0.01**	−0.98	0.13	−7.74	**< 0.0001**
Class 2 (time)	0.11	0.02	5.54	**< 0.0001**	0.29	0.20	1.46	0.14
Shielding	−0.04	0.06	−0.74	0.46	0.72	0.39	1.84	0.07
Community activity	0.16	0.07	2.20	**0.03**	0.40	0.60	0.67	0.50
Leaving home	0.10	0.04	2.42	**0.02**	−0.39	0.27	−1.43	0.15
New work_2	0.07	0.05	1.41	0.16	−0.24	0.32	−0.75	0.45
New work_3	0.14	0.05	2.90	**< 0.01**	−0.07	0.32	−0.23	0.82

*Note:* The values in bold indicate significant results.

When considering predictors influencing trajectory (Table 3), we found that higher levels of well‐being over time were associated with people reporting that they had been engaged in community activities before the pandemic and those who left the house for outdoor exercise or to visit a green space at Wave 1 ( β = 0.16, SE = 0.07, *p* = 0.03 and β = 0.01, SE = 0.04, *p* = 0.02, respectively). We also found slightly higher levels of well‐being in those who were not engaged in paid work, volunteering OR education at Wave 1 but were pre‐pandemic, compared to those who were not ( β = 0.14, SE = 0.05, *p* < 0.01), but not when compared to those who were currently engaged in paid work, volunteering or education at Wave 1 ( β = 0.07, SE = 0.05, *p* = 0.16).

### 2.2 Pandemic Anxiety Scale—LCMM

Table 3 also reports findings from the Pandemic Anxiety Scale. A two‐class model was found to have the best model fit according to the fit indices (see Table S3 and Figure S2). Class membership was 78.50% of the sample for Class 1 and 21.50% of the sample for Class 2 (Table S4). The intercepts were statistically different (with Class 2 being initially higher) but only Class 1 showed a change—a reduction in pandemic anxiety over time ( β = −0.98, SE = 0.13, *p* < 0.0001). Class membership and trajectories were not driven by additional factors other than time (Table 3).

### 2.3 LCMMs for Individual Categorical Outcomes

Tables 4 and 5 present the results from all four single‐item mental health outcomes. Given that response options were deemed categorical, the outputs include parameters relating to thresholds. A threshold model describes the correspondence between each level of an ordinal outcome and the underlying latent process. This means that for a three‐class model, there will be two thresholds to be estimated to split the underlying latent process into three parts (i.e., these are the two boundaries separating Category 1 from 2 and Category 2 from 3). For the question ‘how often do you feel lonely with no one to talk to’, a two‐class model provided the best fit to the data (Table S5 and Figure S3), with 34% of the sample in Class 1 and 66% in Class 2. Each class had significantly different intercepts (with Class 2 having a lower initial category of loneliness than Class 1) and a significant change over time in both classes (Class 1 showing a small reduction in probability of loneliness category, β = −0.15, SE = 0.04, *p* < 0.001; Class 2 also showing a small reduction in probability of loneliness category, β = −0.25, SE = 0.05, *p* < 0.001).

**TABLE 4:** Fixed effects in the class membership models from latent class mixed models (individual item outcomes).

Parameter	Lonely				Anxious				Sad				Angry			
	Coefficient	SE	Wald statistic	*p*	Coefficient	SE	Wald statistic	*p*	Coefficient	SE	Wald statistic	*p*	Coefficient	SE	Wald statistic	*p*
Class 1 (intercept)	**1.48**	**0.60**	**2.47**	0.**01**	**−2.20**	**1.03**	**−2.15**	0.**03**	0.35	0.62	0.57	0.57	**1.28**	**0.60**	**2.14**	0.**03**
Class 2 (intercept)	—	—	—	—	**−6.55**	**2.15**	**−3.05**	**< 0.01**	—	—	—	—	—	—	—	—
Class 1 (shielding)	0.66	0.41	1.60	0.11	1.67	0.88	1.90	0.06	0.71	0.39	1.85	0.06	−0.10	0.39	−0.26	0.80
Class 2 (shielding)	—	—	—	—	**3.32**	**1.02**	**3.26**	**< 0.01**	—	—	—	—	—	—	—	—
Class 1 (community activity)	**−1.17**	**0.41**	**−2.89**	**< 0.01**	**1.62**	**0.57**	**2.85**	**< 0.01**	0.05	0.42	0.12	0.91	−0.60	0.41	−1.46	0.15
Class 2 (community activity)	—	—	—	—	**4.87**	**1.88**	**2.59**	0.**01**	—	—	—	—	—	—	—	—
Class 1 (age)	−0.01	0.01	−0.94	0.35	**0.05**	**0.02**	**2.88**	**< 0.01**	−0.01	0.01	−0.75	0.45	−0.01	0.01	−0.90	0.37
Class 2 (age)	—	—	—	—	**0.04**	**0.02**	**2.18**	0.**03**	—	—	—	—	—	—	—	—
Class 1 (gender)	0.24	0.22	1.11	0.27	−0.68	0.36	−1.92	0.06	**0.90**	**0.22**	**4.11**	**< 0.0001**	0.37	0.21	1.78	0.08
Class 2 (gender)	—	—	—	—	**−1.55**	**0.42**	**−3.68**	**<0.001**	—	—	—	—	—	—	—	—
Class 1 (lives with_2)	**−1.08**	**0.27**	**−3.97**	**< 0.0001**	**1.53**	**0.46**	**3.36**	**< 0.001**	**−0.78**	**0.27**	**−2.85**	**< 0.01**	**−0.60**	**0.27**	**−2.26**	0.**02**
Class 2 (lives with_2)	—	—	—	—	**1.88**	**0.55**	**3.44**	**< 0.001**	—	—	—	—	—	—	—	—
Class 1 (lives with_3)	**−0.86**	**0.30**	**−2.82**	**< 0.01**	0.39	0.45	0.86	0.39	−0.31	0.32	−1.00	0.32	**−0.59**	**0.29**	**−2.02**	0.**04**
Class 2 (lives with_3)	—	—	—	—	0.91	0.53	1.72	0.09	—	—	—	—	—	—	—	—

*Note:* The values in bold indicate significant results.

**TABLE 5:** Fixed effects in the longitudinal models from latent class mixed models (individual item outcomes).

Parameter	Lonely				Anxious				Sad				Angry			
	Coefficient	SE	Wald statistic	*p*	Coefficient	SE	Wald statistic	*p*	Coefficient	SE	Wald statistic	*p*	Coefficient	SE	Wald statistic	*p*
Class 1 (intercept)	—	—	—	—	—	—	—	—	—	—	—	—	—	—	—	—
Class 2 (intercept)	**−1.56**	**0.12**	**−13.53**	**< 0.0001**	**−1.83**	**0.18**	**−10.07**	**< 0.0001**	**−1.68**	**0.12**	**−14.41**	**< 0.0001**	**−1.70**	**0.12**	**−14.34**	**< 0.0001**
Class 3 (intercept)	—	—	—	—	**1.77**	**0.19**	**9.31**	**< 0.0001**	—	—	—	—	—	—	—	—
Class 1 (time)	**−0.15**	**0.04**	**−3.50**	**< 0.001**	**−0.18**	**0.04**	**−5.18**	**< 0.0001**	**−0.17**	**0.04**	**−4.55**	**< 0.0001**	**−0.21**	**0.04**	**−5.65**	**< 0.0001**
Class 2 (time)	**−0.25**	**0.05**	**−4.93**	**< 0.0001**	−0.13	0.09	−1.42	0.16	**−0.10**	**0.05**	**−2.16**	0.**03**	**−0.14**	**0.05**	**−2.81**	0.**01**
Class 3 (time)	—	—	—	—	**−0.32**	**0.09**	**−3.47**	**< 0.001**	—	—	—	—	—	—	—	—
Shielding	−0.14	0.17	−0.83	0.40	**1.12**	**0.18**	**6.34**	**< 0.0001**	0.06	0.13	0.50	0.61	0.24	0.15	1.54	0.12
Community activity	**0.38**	**0.15**	**2.53**	0.**01**	**0.79**	**0.18**	**4.48**	**< 0.0001**	−0.06	0.16	−0.36	0.72	0.20	0.15	1.35	0.18
Leaving home	**−0.22**	**0.10**	**−2.33**	0.**02**	0.02	0.10	0.19	0.85	−0.02	0.09	−0.20	0.84	**−0.21**	**0.09**	**−2.21**	0.**03**
New work_2	**−0.30**	**0.12**	**−2.60**	0.**01**	**−0.26**	**0.12**	**−2.25**	0.**02**	**−0.22**	**0.11**	**−2.05**	0.**04**	−0.15	0.11	−1.43	0.15
New work_3	**−0.27**	**0.11**	**−2.52**	0.**01**	**−0.48**	**0.12**	**−3.92**	**< 0.0001**	**−0.32**	**0.10**	**−3.16**	**< 0.01**	**−0.25**	**0.10**	**−2.46**	0.**01**
Threshold 1	**−1.16**	**0.16**	**−7.22**	**< 0.0001**	−0.26	0.20	−1.27	0.20	**−1.61**	**0.17**	**−9.35**	**< 0.0001**	**−1.41**	**0.16**	**−8.87**	**< 0.0001**
Threshold 2	**1.14**	**0.02**	**51.89**	**< 0.0001**	**1.24**	**0.02**	**58.94**	**< 0.0001**	**1.28**	**0.02**	**59.66**	**< 0.0001**	**1.29**	**0.02**	**59.24**	**< 0.0001**

*Note:* The values in bold indicate significant results.

Class membership (Table 4) was significantly predicted by both who the person lived with (reference category living alone: lives with family, β = 1.08, SE = 0.27, *p* < 0.001; lives with others with intellectual disabilities, β = −0.86, SE = 0.30, *p* < 0.01; those living with family were more likely and those living with others with intellectual disabilities were less likely to be in Class 1 with higher overall levels of loneliness category) and whether they had been active in the community before the pandemic ( β = −1.17, SE = 0.41, *p* < 0.01; those having been active in the community overall being less likely to be in Class 1 with higher overall levels of loneliness).

When considering predictors of the trajectory of loneliness overall across classes (Table 5), the person having been involved in community activity pre‐pandemic increased loneliness ( β = 0.38, SE = 0.15, *p* = 0.01), but loneliness was reduced if participants left the house to exercise or visit green spaces at Wave 1 ( β = −0.22, SE = 0.10, *p* = 0.02) and if they were engaged in paid work/education at Wave 1 or had been in work/education pre‐pandemic ( β = −0.30, SE = 0.12, *p* = 0.01 and β = −0.27, SE = 0.11, *p* = 0.01, respectively).

For the question ‘how often do you feel worried or anxious’, a three‐class model provided the best fit (Supplementary Table S6 and Figure S4), with 66% of the sample in Class 1, 20% in Class 2 and 14% in Class 3. Each class had significantly different intercepts (Class 2 being the lowest initial category, Class 1 in the middle and Class 3 starting with the highest category of worry). There was also a significant change over time in Classes 1 and 3 but not Class 2 (both showing small reductions but Class 3 to a greater extent, Class 1, β = −0.18, SE = 0.04, *p* < 0.0001; Class 3, β = −0.32, SE = 0.09, *p* < 0.001). Class 3 had the greatest reduction from the highest probability of worry category, followed by Class 1, which started from a slightly lower initial position, then Class 2, which had the lowest overall probability of worry category and showed less change over time.

Table 4 shows that class membership was significantly predicted by almost all predictors. Specifically, the likelihood of being in Class 1 compared to being in Class 3 included the following: increased age ( β = 0.05, SE = 0.02, *p* < 0.01); who the person lived with (reference category living alone) (lived with family, β = 1.53, SE = 0.46, *p* < 0.001); and being active in the community before the pandemic ( β = 1.62, SE = 0.57, *p* < 0.01). Those living with family and those active in the community pre‐pandemic were more likely to be in Class 1 compared to Class 3.

The likelihood of being in Class 2 compared to being in Class 3 included the following: shielding ( β = 3.32, SE = 1.02, *p* < 0.01); increased age ( β = 0.04, SE = 0.02, *p* = 0.03); female gender ( β = −1.55, SE = 0.42, *p* < 0.001); who the person lived with (reference category living alone), lived with family ( β = −1.88, SE = 0.55, *p* < 0.001); and being active in the community before the pandemic ( β = 4.87, SE = 1.88, *p* = 0.01). Those living with family and those active in the community pre‐pandemic were more likely to be in Class 2 compared to class 3.

The predictors of change in worry overall across classes (Table 5) included whether the person had been involved in community activity pre‐pandemic or was shielding at Wave 1, with both being associated with increasing worry over time ( β = 1.12, SE = 0.18, *p* < 0.0001 and β = 0.79, SE = 0.18, *p* < 0.0001, respectively), but worry was reduced if the person was leaving the house to exercise or visit green spaces at Wave 1 ( β = −0.22, SE = 0.10, *p* = 0.02) and if they were engaged in paid work/education or had been in work/education pre‐pandemic ( β = −0.26, SE = 0.12, *p* = 0.02 and β = −0.48, SE = 0.12, *p* < 0.0001, respectively).

For the question ‘how often do you feel sad or down’, a two‐class model provided the best fit (Table S7 and Figure S5), with 56% of the sample in Class 1 and 44% in Class 2. We found a significant change over time in both classes (Class 1 showing a reduction in probability of sadness category, β = − 0.17, SE = 0.04, *p* < 0.0001; Class 2 showing a smaller reduction, β = −0.10, SE = 0.05, *p* = 0.03) and a lower probability of sadness initially ( β = −1.68, SE = 0.12, *p* < 0.0001).

Class membership (Table 4) was significantly predicted by who the person lived with (reference category living alone: lived with family, β = −0.78, SE = 0.27, *p* < 0.01) and if they were female ( β = 0.90, SE = 0.22, *p* < 0.0001, with both more likely to be in Class 1 with steeper reduction in sadness). Regarding predictors of sadness trajectory, only if they were engaged in paid work/education at Wave 1 or had been in work/education pre‐pandemic ( β = − 0.22, SE = 0.11, *p* = 0.04 and β = −0.32, SE = 0.10, *p* < 0.01, respectively) were found to be statistically significant. Those in work prior to the pandemic or at Wave 1 reported reduced sadness over time.

For the question ‘how often do you feel angry or frustrated’, a two‐class model provided the best fit (Table S8 and Figure S6), with 55% of the sample in Class 1 and 45% in Class 2. We found a significant change over time in both classes (Class 1 showing a reduction probability in the anger category, β = −0.21, SE = 0.04, *p* < 0.0001; Class 2 showing a smaller reduction, β = −0.14, SE = 0.05, *p* = 0.01) and a lower probability of anger initially ( β = −1.70, SE = 0.12, *p* < 0.0001).

Class membership (Table 4) was significantly predicted only by who the person lived with (reference category living alone: lived with family, β = −0.60, SE = 0.27, *p* = 0.02; lives with others with intellectual disabilities, β = −0.59, SE = 0.29, *p* = 0.04); both those living with family or with others with intellectual disabilities were less likely to be in Class 1—with steeper reductions in anger. Longitudinal model predictors (overall across classes) showed reductions in anger when participants had been in work/education pre‐pandemic (*β* = −0.25, SE = 0.10, *p* = 0.01) and when they were leaving the house to exercise or visit green spaces at Wave 1 ( β = −0.21, SE = 0.09, *p* = 0.03).

#### 2.3.1 Class Summary Descriptives Across All Models

Table 6 presents the descriptive statistics (means, SD and *N*) for outcomes by time and class. This reflects the model information for intercepts and change over time.

**TABLE 6:** Descriptives summaries (mean, SD and *N*) across outcomes by time and class.

Outcome	Class	Wave				
		1	1	2	3	4
		*n*	Mean (SD)	Mean (SD)	Mean (SD)	Mean (SD)
WEMWBS	1	372	3.32 (0.41)	3.32 (0.50)	3.27 (0.52)	3.15 (0.51)
	2	224	2.34 (0.40)	2.49 (0.44)	2.51 (0.49)	2.76 (0.57)
PAS total score	1	454	13.5 (3.70	10.6 (3.02)	10.3 (2.85)	10.7 (2.83)
	2	135	15.4 (3.75)	13.9 (3.66)	14.8 (3.19)	16.9 (2.59)
Lonely	1	248	2.33 (0.66)	2.16 (0.66)	2.13 (0.66)	2.03 (0.74)
	2	348	1.34 (0.55)	1.30 (0.54)	1.14 (0.38)	1.12 (0.33)
Anxious	1	394	2.07 (0.70)	1.91 (0.66)	1.82 (0.69)	1.74 (0.67)
	2	119	1.32 (0.57)	1.23 (0.45)	1.24 (0.51)	1.28 (0.57)
	3	83	2.84 (0.37)	2.68 (0.58)	2.68 (0.53)	2.38 (0.66)
Sad	1	333	2.35 (0.57)	2.25 (0.61)	2.15 (0.61)	2.04 (0.66)
	2	263	1.38 (0.54)	1.27 (0.46)	1.24 (0.43)	1.33 (0.47)
Angry	1	325	2.35 (0.59)	2.11 (0.58)	2.11 (0.60)	1.94 (0.64)
	2	271	1.38 (0.54)	1.23 (0.46)	1.21 (0.42)	1.26 (0.62)

#### 2.3.2 Sensitivity Analysis for Missingness

Given that missing data were present in both outcome (up to 22%) and predictor variables (up to 8%), we conducted a missing‐data sensitivity analysis using multiple imputation (see Tables S9 and S10). For most outcomes, results were approximately in agreement with complete case analyses, with some reduction in effect sizes. Notable differences occurred for the Pandemic Anxiety Scale outcome analysis. Size of class was inconsistent across imputed data sets analysed, so pooling was problematic, leading to estimates that were markedly different. This is likely due to no predictors being significant in either part of the model and very little change over time. For individual item outcomes, estimates for class membership were generally consistent in direction but differed in magnitude, potentially due to the sensitive nature of mixture models to additional data.

## 3 Discussion

A longitudinal research design was used to model the trajectories of mental health and well‐being across the pandemic. Both mental well‐being and pandemic anxiety showed relatively little change over time according to overall sample summary statistics. Four single‐item outcome measures of loneliness, anxiety, sadness and anger showed more consistent reduction over time as pandemic restrictions gradually eased, although we acknowledge that the probability of change in categorical response is potentially less precise than a continuous measure. While comparative longitudinal data are not available for people with intellectual disabilities, research with autistic adults showed that levels of loneliness and stress remained relatively stable across the pandemic (Scheeren et al. 2022). Given that almost all restrictions had been lifted by Wave 4 of the study, it is likely that this general improvement in mental health in our study was linked to the easing of pandemic restrictions. However, many people with intellectual disabilities continued to report experiences of anxiety, loneliness, sadness and anger/frustration.

These overall patterns of change masked some trajectory subgroups that give a fuller picture of changing well‐being and mental health for adults with intellectual disabilities through the pandemic period. In terms of well‐being, most adults with intellectual disabilities showed relatively high levels of well‐being but a small reduction over time; around 38% had lower initial levels of well‐being but with a small increase over time. The small reduction in well‐being across waves in one of the groups might be explained by the initial higher levels of well‐being in this group. This initial higher well‐being appeared to be related to greater levels of social support pre‐pandemic within this group, and the COVID pandemic then had a negative impact on social support/social connections. The vast majority also had reduced pandemic anxiety over time, but a significant subgroup (21%) had higher levels of pandemic anxiety that did not reduce. For pandemic anxiety, it is important to be cautious about these findings since they were sensitive to missing data.

For each single‐item mental health outcome, there was at least one subgroup of adults with intellectual disabilities who were at higher risk of reporting problems. For loneliness, one‐third of the sample had a higher likelihood of loneliness but also were gradually less likely over time to report being lonely. For worry, 14% had the highest chance of reporting worry throughout but also the steepest reduction, whereas two‐thirds had a middling chance of worry, but this did not change over time. For sadness and anger, just over half the sample (56% and 55%, respectively) had higher chances of reporting problems across the study period, although their sadness and anger did reduce more steeply over time.

In terms of predictors of trajectory/class membership and predictors of overall change in outcomes, there was some consistency in relation to key findings, but generally, different factors were influential for class membership and overall change across time. The exception was pre‐pandemic involvement in community activities. Those involved in community activities pre‐pandemic were less likely to be in trajectory classes with high chances of loneliness and worry and also had higher levels of well‐being over time. However, those involved in community activities pre‐pandemic were more likely to report increasing loneliness and worry over time. Previous qualitative research found that a loss of engagement in meaningful activities, due to pandemic restrictions, negatively impacted the mental well‐being of people with intellectual disabilities (Parchomiuk 2024). This study supported these findings. These latter associations may be related to a sharper sense of comparison (i.e., missing activities that were previously enjoyed).

For trajectory classes, living with family (compared to living alone) and living with other people with intellectual disabilities (compared to living alone) were both associated with subgroups having higher overall well‐being and lower anger. Living with family was also associated with trajectory subgroup membership having lower levels of anger and worry. However, living with family was associated with the trajectory group more likely to report sadness (though reduction in sadness was also steeper in this trajectory class). A contrasting pattern was found for loneliness where those living with family were more likely to be in the minority high‐loneliness group and those living with other people with intellectual disabilities were less likely to be in this group. It may be that connections to other people were more numerous or potentially more supportive when adults with intellectual disabilities were living with other people with intellectual disabilities as opposed to with family. Our research aligns with findings of a large‐scale survey with adults with intellectual disabilities during the first lockdown of the pandemic (May 2020), where greater levels of social support were predictive of lower levels of depression and anxiety and higher well‐being (Essau and De la Torre‐Loque 2021). While our findings highlighted the importance of social connection in maintaining people's well‐being, our analysis was limited to data on who people with intellectual disabilities lived with during the pandemic. Further research looking at the social support networks of people with intellectual disabilities and associations between models of social support and the mental health and well‐being of people with intellectual disabilities will be valuable in informing future support. Considering general patterns of change across the data collection waves, adults with intellectual disabilities who left the house to exercise or to visit green spaces reported increased well‐being and reduced loneliness, worry and anger. This aligns with the findings of a systematic review conducted by Diz et al. (2024), which found a significant association between an engagement in physical exercise and higher emotional well‐being in people with intellectual disabilities. Engagement in work, volunteering or education pre‐pandemic and/or at Wave 1 was also associated with reduced loneliness, worry and sadness. Pre‐pandemic engagement in work, volunteering or education pre‐pandemic was additionally associated with increased well‐being and reduced anger over time.

### 3.1 Strengths and Limitations

As far as we are aware, these data are unique in relation to understanding patterns of well‐being, pandemic anxiety and mental health for adults with intellectual disabilities over multiple time points during the COVID‐19 pandemic period. Thus, it is difficult to compare the findings with other research, and it is unclear if there is an opportunity for replication of these longitudinal analyses in other samples. Obtaining longitudinal self‐report data from people with intellectual disabilities is a strength of this research and provides a more nuanced account of factors that affected their mental health across the pandemic period. Careful consideration was exercised to capture the views and experiences of adults with intellectual disabilities in a valid and reliable way and minimise the risk of socially desirable responses (Kooijmans et al. 2022). However, a significant limitation of the study is the absence of pre‐pandemic data about the participants' mental health and well‐being. This lack of pre‐pandemic data limits the ability to draw conclusions on the *impact* of the pandemic on the participants' mental health. In addition, the participants who took part in this study were largely recruited through third‐sector and service provider organisations and included few people from UK ethnic minority groups. Hence, this sample may not be completely representative and may have excluded certain subgroups.

### 3.2 Policy and Practical Implications

The present study highlighted that having purposeful activities, exercising and spending time outdoors were associated with better outcomes for the mental health and well‐being of people with intellectual disabilities during the COVID‐19 pandemic. The findings also identified factors which reduced people's resilience to the restricted lives that they led during the pandemic (e.g., those not in work, volunteering or education pre‐pandemic).

In addition to response to future pandemics, these findings also have relevance to the current crisis in social care in the UK and internationally (Curry and Oung 2021). The findings highlight that social connection and engagement in purposeful activity are vital to maintaining people with intellectual disabilities' mental health and well‐being.

## Ethics Statement

The Manchester Metropolitan University Faculty of Health, Psychology and Social Care Faculty Research Ethics Committee provided research ethical review and approval for this study.

## Conflicts of Interest

The authors declare no conflicts of interest.

## Supporting information


**Table S1.** Model fit indices for main analysis models with Classes 1–5.
**Table S2.** Class membership percentages for main analysis models with Classes 1–5.
**Figure S1.** Elbow plots of model fit indices for main analysis models with Classes 1–5.
**Table S3.** Model fit indices for main analysis models with Classes 1–5.
**Table S4.** Class membership percentages for main analysis models with Classes 1–5.
**Figure S2.** Elbow plots of model fit indices for base models with Classes 1–5.
**Table S5.** Model fit indices for main analysis models with Classes 1–5.
**Figure S3.** Elbow plots of model fit indices for main analysis models with Classes 1–5.
**Table S6.** Model fit indices for main analysis models with Classes 1–5.
**Figure S4.** Elbow plots of model fit indices for main analysis models with Classes 1–5.
**Table S7.** Model fit indices for main analysis models with Classes 1–5.
**Figure S5.** Elbow plots of model fit indices for main analysis models with Classes 1–5.
**Table S8.** Model fit indices for main analysis models with Classes 1–5.
**Figure S6.** Elbow plots of model fit indices for main analysis models with Classes 1–5.
**Table S9.** Imputed datasets pooled estimates (two‐class model) for WEMWBS and PAS outcomes.
**Table S10.** Imputed datasets pooled estimates for individual item outcomes: lonely, anxious, sad and angry.

## Data Availability

A quantitative dataset will be archived online in a form that will be available to researchers after all waves of data collection for the project have been completed.
