# Supplementary material for: The Mental Health and Well‐Being of Adults With Intellectual Disabilities During the COVID‐19 Pandemic Across the UK: A Four‐Wave Longitudinal Analysis
Source: J Intellect Disabil Res. 2025 Jul 15;69(10):1132–42. doi: 10.1111/jir.70006 (PMC12576364; doi:10.1111/jir.70006)
Supplement: Supplementary file 1 — Table S1. Model fit indices for main analysis models with Classes 1–5. Table S2. Class membership percentages for main analysis models with Classes 1–5. Figure S1. Elbow plots of model fit indices for main analysis models with Classes 1–5. Table S3. Model fit indices for main analysis models with Classes 1–5. Table S4. Class membership percentages for main analysis models with Classes 1–5. Figure S2. Elbow plots of model fit indices for base models with Classes 1–5. Table S5. Model fit indices for main analysis models with Classes 1–5. Figure S3. Elbow plots of model fit indices for main analysis models with Classes 1–5. Table S6. Model fit indices for main analysis models with Classes 1–5. Figure S4. Elbow plots of model fit indices for main analysis models with Classes 1–5. Table S7. Model fit indices for main analysis models with Classes 1–5. Figure S5. Elbow plots of model fit indices for main analysis models with Classes 1–5. Table S8. Model fit indices for main analysis models with Classes 1–5. Figure S6. Elbow plots of model fit indices for main analysis models with Classes 1–5. Table S9. Imputed datasets pooled estimates (two‐class model) for WEMWBS and PAS outcomes. Table S10. Imputed datasets pooled estimates for individual item outcomes: lonely, anxious, sad and angry. [file JIR-69-1132-s001.docx]

**Supplementary material**

# **1. WEMWBS (mean)**

***1.1 Main analysis model – model including covariates in the class and longitudinal models***

**Table S1.** Model fit indices for main analysis models with classes 1-5.

|  |  |  |  |  |  |  |  |  |
| --- | --- | --- | --- | --- | --- | --- | --- | --- |
| **No. of classes** | **log likelihood** | **Number of parameters** | **AIC** | **BIC** | **SABIC** | **Entropy** | **ICL1** | **ICL2** |
| 1 | -1454.67 | 11 | 2931.34 | 2979.84 | 2944.92 | 1.00 | 2979.84 | 2979.84 |
| 2 | -1385.05 | 20 | 2810.09 | 2897.80 | 2834.31 | 0.63 | 3051.81 | 3051.44 |
| 3 | -1378.17 | 29 | 2814.34 | 2941.51 | 2849.45 | 0.63 | 3180.22 | 3177.42 |
| 4 | -1368.86 | 38 | 2813.73 | 2980.36 | 2859.73 | 0.63 | 3285.15 | 3286.81 |
| 5 | -1351.10 | 47 | 2796.21 | 3002.31 | 2853.10 | 0.69 | 3296.51 | 3285.52 |
|  |  |  |  |  |  |  |  |  |

**Table S2.** Class membership percentages for main analysis models with classes 1-5.

|  |  |  |  |  |
| --- | --- | --- | --- | --- |
| **Class 1 (%)** | **Class 2 (%)** | **Class 3 (%)** | **Class 4 (%)** | **Class 5 (%)** |
| 100.00 |  |  |  |  |
| 62.23 | 37.77 |  |  |  |
| 59.70 | 35.24 | 5.06 |  |  |
| 21.92 | 14.33 | 57.00 | 6.75 |  |
| 15.18 | 15.51 | 29.34 | 11.47 | 28.50 |

**Figure S1.** Elbow plots of model fit indices for main analysis models with classes 1-5.


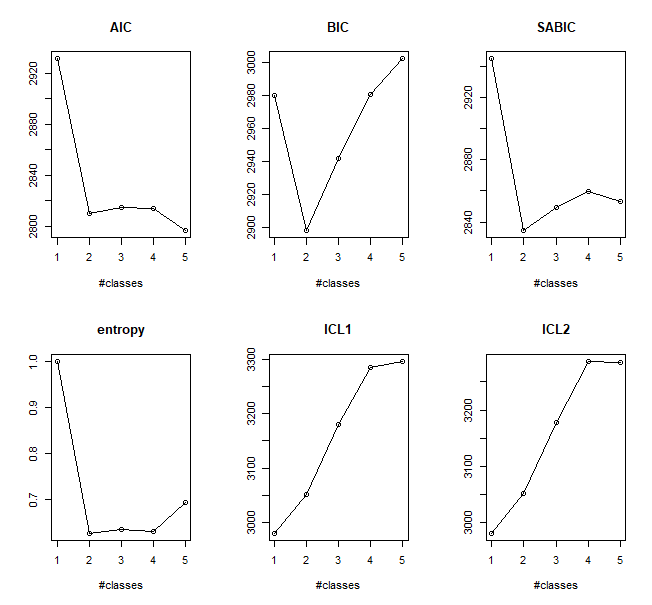


# **2. Pandemic anxiety scale (total score)**

***2.1 Main analysis model – model including covariates in the class and longitudinal models***

**Table S3.** Model fit indices for main analysis models with classes 1-5.

|  |  |  |  |  |  |  |  |  |
| --- | --- | --- | --- | --- | --- | --- | --- | --- |
| **No. of classes** | **log likelihood** | **Number of parameters** | **AIC** | **BIC** | **SABIC** | **Entropy** | **ICL1** | **ICL2** |
| 1 | -4786.09 | 11 | 9594.19 | 9642.56 | 9607.63 | 1.00 | 9642.56 | 9642.56 |
| 2 | -4665.44 | 20 | 9370.87 | 9458.34 | 9394.84 | 0.45 | 9681.16 | 9704.18 |
| 3 | -4655.51 | 29 | 9369.01 | 9495.84 | 9403.77 | 0.68 | 9701.16 | 9711.46 |
| 4 | -4644.73 | 38 | 9365.45 | 9531.64 | 9411.00 | 0.72 | 9755.04 | 9758.40 |
| 5 | - | 47 | - | - | - | - | - | - |

**Table S4.** Class membership percentages for main analysis models with classes 1-5.

|  |  |  |  |  |
| --- | --- | --- | --- | --- |
| **Class 1 (%)** | **Class 2 (%)** | **Class 3 (%)** | **Class 4 (%)** | **Class 5 (%)** |
| 100.00 |  |  |  |  |
| 78.50 | 21.50 |  |  |  |
| 76.28 | 17.75 | 5.97 |  |  |
| 14.85 | 22.01 | 55.63 | 7.51 |  |
| 0.00 | 0.00 | 0.00 | 0.00 | 0.00 |
|  |  |  |  |  |

**Figure S2.** Elbow plots of model fit indices for base models with classes 1-5.


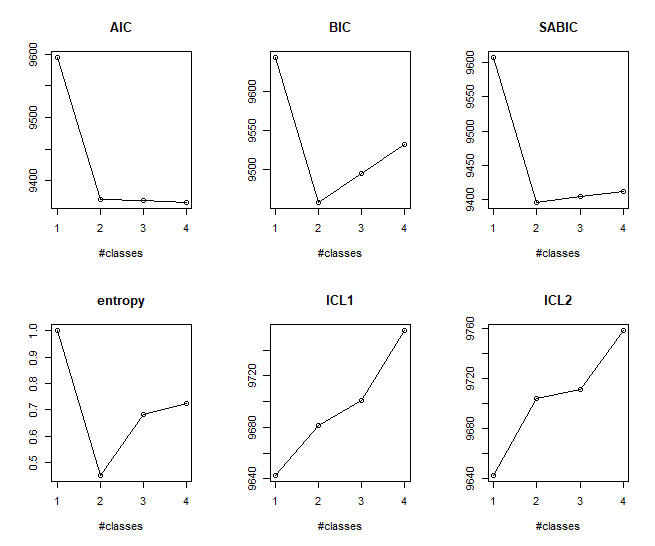


# **3. Individual item outcome (How often do you feel lonely with no one to talk to?)**

***3.1 Main analysis model – model including covariates in the class and longitudinal models***

**Table S5.** Model fit indices for main analysis models with classes 1-5.

|  |  |  |  |  |  |  |  |  |
| --- | --- | --- | --- | --- | --- | --- | --- | --- |
| **No. of classes** | **log likelihood** | **Number of parameters** | **AIC** | **BIC** | **SABIC** | **Entropy** | **ICL1** | **ICL2** |
| 1 | -1863.21 | 7 | 3740.41 | 3771.27 | 3749.05 | 1.00 | 3771.27 | 3771.27 |
| 2 | -1691.61 | 15 | 3413.22 | 3479.15 | 3431.53 | 0.66 | 3621.78 | 3624.20 |
| 3 | -1675.10 | 23 | 3396.20 | 3497.29 | 3424.27 | 0.67 | 3714.76 | 3716.38 |
| 4 | -1659.34 | 31 | 3380.68 | 3516.94 | 3418.52 | 0.63 | 3823.19 | 3845.04 |
| 5 | -1655.98 | 39 | 3389.96 | 3561.38 | 3437.57 | 0.62 | 3925.21 | 3960.23 |
|  |  |  |  |  |  |  |  |  |

**Figure S3.** Elbow plots of model fit indices for main analysis models with classes 1-5.


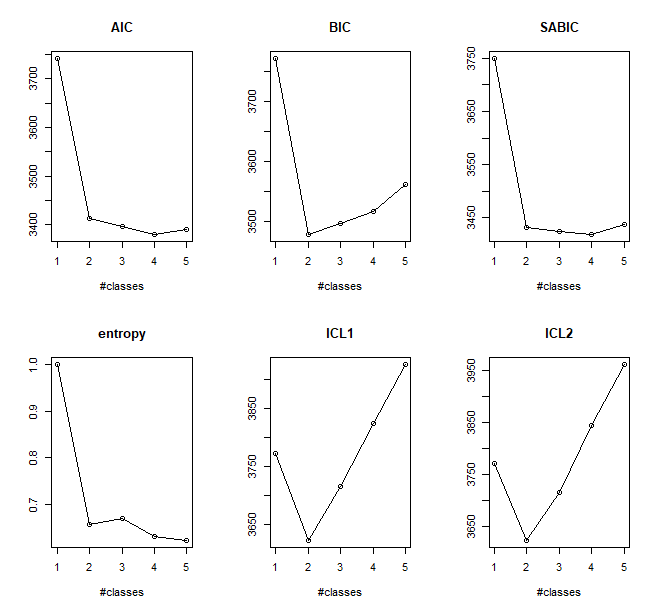


# **4. Individual item outcome (how often do you feel worried or anxious?)**

***4.1 Main analysis model – model including covariates in the class and longitudinal models***

**Table S6.** Model fit indices for main analysis models with classes 1-5.

|  |  |  |  |  |  |  |  |  |
| --- | --- | --- | --- | --- | --- | --- | --- | --- |
| **No. of classes** | **log likelihood** | **Number of parameters** | **AIC** | **BIC** | **SABIC** | **Entropy** | **ICL1** | **ICL2** |
| 1 | -2013.1 | 8 | 4042.20 | 4077.47 | 4052.07 | 1.00 | 4077.47 | 4077.47 |
| 2 | -1847.36 | 17 | 3728.72 | 3803.27 | 3749.30 | 0.64 | 3950.10 | 3949.95 |
| 3 | -1808.66 | 26 | 3669.31 | 3783.33 | 3700.79 | 0.72 | 3965.67 | 3966.21 |
| 4 | -1798.35 | 35 | 3666.70 | 3820.18 | 3709.06 | 0.70 | 4064.56 | 4066.09 |
| 5^✝^ | -1787.72 | 44 | 3663.44 | 3856.39 | 3716.70 | 0.76 | 4085.75 | 4094.35 |

^✝^Convergence was not achieved for this model

**Figure S4.** Elbow plots of model fit indices for main analysis models with classes 1-5.

#
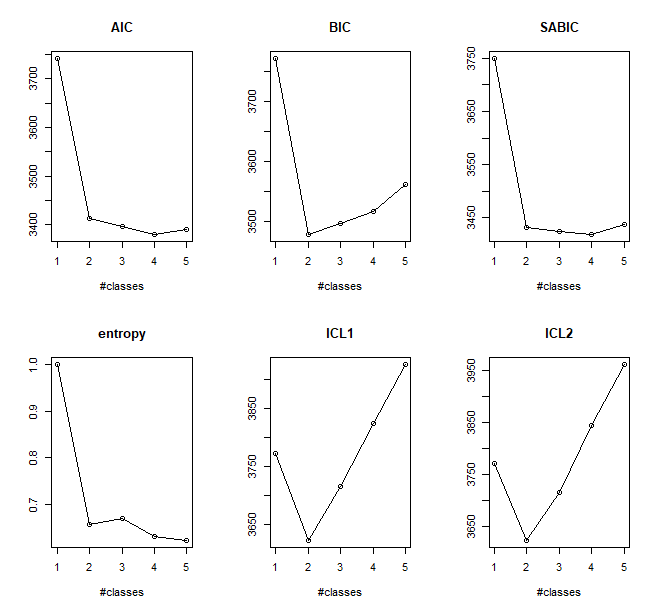


# **5. Individual item outcome (how often did you feel sad or down?)**

|  |  |  |  |  |
| --- | --- | --- | --- | --- |

***5.1 Main analysis model – model including covariates in the class and longitudinal models***

**Table S7.** Model fit indices for main analysis models with classes 1-5.

|  |  |  |  |  |  |  |  |  |
| --- | --- | --- | --- | --- | --- | --- | --- | --- |
| **No. of classes** | **log likelihood** | **Number of parameters** | **AIC** | **BIC** | **SABIC** | **Entropy** | **ICL1** | **ICL2** |
| 1 | -2013.10 | 8 | 4042.20 | 4077.47 | 4052.07 | 1.00 | 4077.47 | 4077.47 |
| 2 | -1766.62 | 17 | 3567.25 | 3641.80 | 3587.83 | 0.63 | 3795.60 | 3796.04 |
| 3 | -1751.94 | 26 | 3555.87 | 3669.89 | 3587.35 | 0.59 | 3934.00 | 3965.46 |
| 4 | -1740.27 | 35 | 3550.53 | 3704.02 | 3592.90 | 0.66 | 3981.68 | 3986.07 |
| 5 | -1727.30 | 44 | 3542.60 | 3735.55 | 3595.86 | 0.72 | 3998.60 | 3999.04 |

**Figure S5.** Elbow plots of model fit indices for main analysis models with classes 1-5.


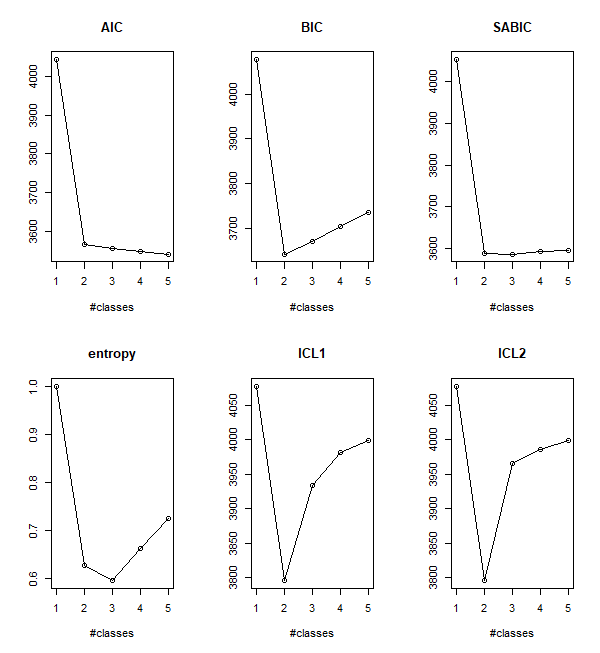


# **6. Individual item outcome (how often did you feel angry or frustrated?)**

***6.2 Main analysis model – model including covariates in the class and longitudinal models***

**Table S8.** Model fit indices for main analysis models with classes 1-5.

| **No. of classes** | **log likelihood** | **Number of parameters** | **AIC** | **BIC** | **SABIC** | **Entropy** | **ICL1** | **ICL2** |
| --- | --- | --- | --- | --- | --- | --- | --- | --- |
| 1 | -1921.03 | 8 | 3858.07 | 3893.33 | 3867.94 | 1.00 | 3893.33 | 3893.33 |
| 2 | -1751.49 | 17 | 3536.98 | 3611.53 | 3557.56 | 0.62 | 3766.34 | 3761.32 |
| 3 | -1738.84 | 26 | 3529.67 | 3643.69 | 3561.15 | 0.57 | 3926.48 | 3954.07 |
| 4 | -1724.57 | 35 | 3519.14 | 3672.62 | 3561.51 | 0.69 | 3928.90 | 3937.28 |
| 5 | -1712.08 | 44 | 3512.17 | 3705.12 | 3565.43 | 0.69 | 4002.11 | 4036.31 |
|  |  |  |  |  |  |  |  |  |

**Figure S6.** Elbow plots of model fit indices for main analysis models with classes 1-5.


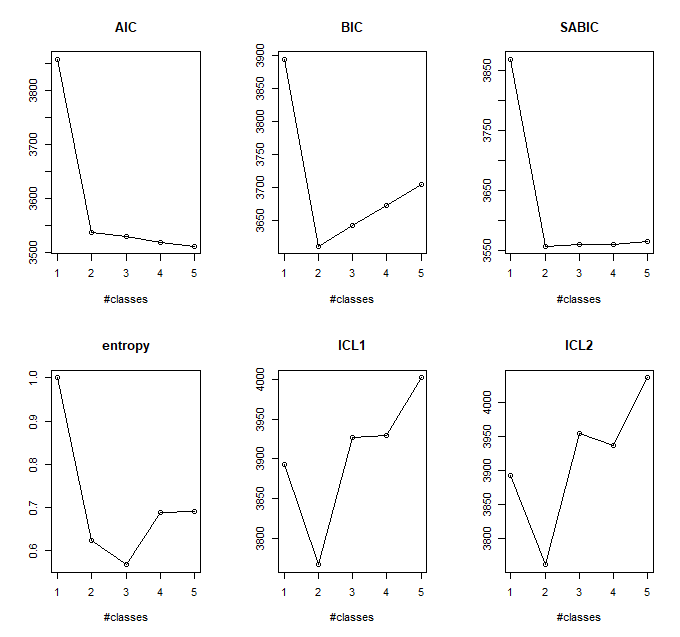


# **7. Missing data sensitivity analyses**

**Table S9**. Imputed datasets pooled estimates (2 class model) for WEMWBS and PAS outcomes

|  |  |  |  |  |
| --- | --- | --- | --- | --- |
|  | **WEMWBS (mean score)** | | **PAS** | |
| **Parameter** | **Coefficient** | **Std error** | **Coefficient** | **Std error** |
| **Fixed effects in the class-membership model** |  |  |  |  |
| class1 (intercept) | -0.88 | 0.018 | -4.76 | 5.971 |
| class1 (shielding) | -0.01 | 0.009 | -0.51 | 0.066 |
| class1 (community action) | -0.37 | 0.011 | 5.32 | 5.970 |
| class1 (Age) | 0.03 | 0.000 | -0.04 | 0.004 |
| class1 (Gender) | -0.71 | 0.006 | -0.40 | 0.039 |
| class1 (lives with_2) | 1.05 | 0.007 | 0.98 | 0.050 |
| class1 (lives with_3) | 1.20 | 0.008 | 0.65 | 0.061 |
| **Fixed effects in the longitudinal model** |  |  |  |  |
| class1 (intercept) | 3.06 | 0.002 | 14.14 | 0.028 |
| class2 (intercept) | 2.25 | 0.002 | 13.28 | 0.016 |
| class1 (time) | -0.07 | 0.000 | -0.38 | 0.010 |
| class2 (time) | 0.11 | 0.001 | -0.19 | 0.005 |
| Shielding | -0.03 | 0.001 | 0.66 | 0.010 |
| Community activity | 0.11 | 0.001 | -0.16 | 0.013 |
| Leaving home | 0.10 | 0.001 | -0.44 | 0.008 |
| New work_2 | 0.09 | 0.001 | -0.41 | 0.009 |
| New work_3 | 0.11 | 0.001 | -0.08 | 0.009 |
|  |  |  |  |  |

**Table S10**. Imputed datasets pooled estimates for individual item outcomes: Lonely, Anxious, Sad, and Angry.

|  | **Lonely** | | **Anxious** | | **Sad** | | **Angry** | |
| --- | --- | --- | --- | --- | --- | --- | --- | --- |
| **Parameter** | **Coefficient** | **Std error** | **Coefficient** | **Std error** | **Coefficient** | **Std error** | **Coefficient** | **Std error** |
| **Fixed effects in the class-membership model** |  |  |  |  |  |  |  |  |
| class1 (intercept) | 0.09 | 0.074 | 1.96 | 0.189 | 0.60 | 0.066 | 1.26 | 0.016 |
| class2 (intercept) | - | - | 2.30 | 0.196 | - | - | - | - |
| class1 (Shielding) | 0.03 | 0.046 | -0.29 | 0.049 | 0.11 | 0.040 | 0.13 | 0.009 |
| class2 (Shielding) | - | - | 0.33 | 0.081 | - | - | - | - |
| class1 (community activity) | 0.06 | 0.055 | -0.70 | 0.177 | -0.26 | 0.047 | -0.56 | 0.011 |
| class2 (community activity) | - | - | -1.00 | 0.183 | - | - | - | - |
| class1 (age) | -0.01 | 0.001 | 0.01 | 0.001 | 0.00 | 0.001 | -0.01 | 0.000 |
| class2 (age) | - | - | -0.02 | 0.001 | - | - | - | - |
| class1 (gender) | 0.16 | 0.022 | 0.64 | 0.038 | 0.58 | 0.023 | 0.41 | 0.005 |
| class2 (gender) | - | - | 1.12 | 0.031 | - | - | - | - |
| class1 (lives with2) | -0.72 | 0.029 | 0.17 | 0.028 | -0.46 | 0.028 | -0.59 | 0.006 |
| class2 (lives with2) | - | - | -0.75 | 0.032 | - | - | - | - |
| class1 (lives with3) | -0.42 | 0.031 | -0.10 | 0.029 | -0.15 | 0.032 | -0.69 | 0.007 |
| class2 (lives with3) | - | - | -0.55 | 0.032 | - | - | - | - |
| **Fixed effects in the longitudinal model** |  |  |  |  |  |  |  |  |
| class1 (intercept) | - | - | - | - | - | - | - | - |
| class2 (intercept) | -0.81 | 0.012 | 0.97 | 0.019 | -0.79 | 0.011 | -1.38 | 0.003 |
| class3 (intercept) | - | - | -1.46 | 0.136 | - | - | - | - |
| class1 (time) | -0.15 | 0.004 | -0.09 | 0.002 | -0.11 | 0.003 | -0.19 | 0.001 |
| class2 (time) | -0.10 | 0.004 | -0.27 | 0.007 | -0.05 | 0.004 | -0.020 | 0.001 |
| class3 (time) | - | - | 0.14 | 0.059 | - | - | - | - |
| Shielding | 0.10 | 0.013 | 0.18 | 0.012 | 0.11 | 0.010 | 0.15 | 0.002 |
| Community activity | -0.06 | 0.015 | 0.13 | 0.007 | 0.08 | 0.012 | 0.12 | 0.003 |
| Leaving home | -0.13 | 0.007 | -0.08 | 0.004 | -0.07 | 0.006 | -0.13 | 0.001 |
| New work2 | -0.15 | 0.008 | -0.19 | 0.005 | -0.20 | 0.007 | -0.17 | 0.002 |
| New work3 | -0.19 | 0.008 | -0.29 | 0.005 | -0.28 | 0.007 | -0.18 | 0.002 |
| Threshold 1 | -0.87 | 0.019 | 0.65 | 0.011 | -1.00 | 0.015 | -1.16 | 0.003 |
| Threshold 2 | 1.08 | 0.002 | 1.16 | 0.001 | 1.22 | 0.002 | 1.22 | 0.000 |
|  |  |  |  |  |  |  |  |  |
